# Seroepidemiology of Lassa virus in pregnant women in Southern Nigeria: A prospective hospital-based cohort study

**DOI:** 10.1371/journal.pntd.0011354

**Published:** 2023-05-22

**Authors:** Nzelle Delphine Kayem, Sylvanus Okogbenin, Joseph Okoeguale, Mojeed Momoh, Antonia Njoku, Reuben Eifediyi, Xavier Enodiana, Hilary Ngwu, Wilfred Irhiogbe, Yemisi Ighodalo, Thomas Olokor, George Odigie, Lyndsey Castle, Sophie Duraffour, Lisa Oestereich, Prabin Dahal, Proochista Ariana, Stephan Gunther, Peter Horby

**Affiliations:** 1 Nuffield Department of Medicine, University of Oxford, Oxford, United Kingdom; 2 Department of Obstetrics and Gynaecology, Irrua Specialist Teaching Hospital, Irrua, Nigeria; 3 Institute of Lassa fever Research and Control, Irrua Specialist Teaching Hospital, Irrua, Nigeria; 4 Bernhard-Nocht-Institute for Tropical Medicine, Hamburg, Germany; NIAID Integrated Research Facility, UNITED STATES

## Abstract

**Background:**

There is limited epidemiological evidence on Lassa fever in pregnant women with acute gaps on prevalence, infection incidence, and risk factors. Such evidence would facilitate the design of therapeutic and vaccine trials and the design of control programs. Our study sought to address some of these gaps by estimating the seroprevalence and seroconversion risk of Lassa fever in pregnant women.

**Methodology/Principal findings:**

We conducted a prospective hospital-based cohort between February and December 2019 in Edo State, Southern Nigeria, enrolling pregnant women at antenatal clinic and following them up at delivery. Samples were evaluated for IgG antibodies against Lassa virus. The study demonstrates a seroprevalence of Lassa IgG antibodies of 49.6% and a seroconversion risk of 20.8%. Seropositivity was strongly correlated with rodent exposure around homes with an attributable risk proportion of 35%. Seroreversion was also seen with a seroreversion risk of 13.4%.

**Conclusions/Significance:**

Our study suggests that 50% of pregnant women were at risk of Lassa infection and that 35.0% of infections might be preventable by avoiding rodent exposure and conditions which facilitate infestation and the risk of human-rodent contact. While the evidence on rodent exposure is subjective and further studies are needed to provide a better understanding of the avenues of human-rodent interaction; public health measures to decrease the risk of rodent infestation and the risk of spill over events may be beneficial. With an estimated seroconversion risk of 20.8%, our study suggests an appreciable risk of contracting Lassa fever during pregnancy and while most of these seroconversions may not be new infections, given the high risk of adverse outcomes in pregnancy, it supports the need for preventative and therapeutic options against Lassa fever in pregnancy. The occurrence of seroreversion in our study suggests that the prevalence obtained in this, and other cohorts may be an underestimate of the actual proportion of women of childbearing age who present at pregnancy with prior LASV exposure. Additionally, the occurrence of both seroconversion and seroreversion in this cohort suggests that these parameters would need to be considered for the development of Lassa vaccine efficacy, effectiveness, and utility models.

## Author summary

Lassa virus is a viral haemorrhagic fever endemic to West Africa and yet to date there is limited information on its epidemiology in pregnancy. Information obtained from epidemiology studies is important because such information facilitates the design and costing of disease control programs and the design of clinical trials. Our study evaluated the epidemiology of Lassa fever in pregnant women specifically we looked at the prevalence, incidence, and risk factors for infection in pregnant women in South Nigeria. We found that 49.6% of pregnant women had antibodies to Lassa virus suggesting about 50% of pregnant women were at risk of Lassa infection. Additionally, while, the evidence was not conclusive, we found an appreciable risk of contracting Lassa fever during pregnancy. Given that, Lassa fever has high case fatality in pregnancy, our data supports the need for vaccines and drugs against Lassa fever. Our study also suggests that 35.0% of infections might be preventable by avoiding rodent exposure and conditions which facilitate rodent infestation and the risk of human-rodent contact. This suggests that public health measures such as proper sanitation and hygiene, improved housing and health education may be beneficial in reducing the risk of infection.

## Introduction

Lassa fever is a zoonotic infection endemic to the West African subregion where cases are reported throughout the year, with peak incidences between November and May in endemic countries. [1–3] Modelling studies in Sub-Saharan Africa suggest that about 37.7 million people in 14 West African countries are at risk of infection [4] with an estimated 5,000 to 60,000 deaths annually. [5, 6]

Pregnant women and their foetuses are particularly prone to adverse outcomes from Lassa virus infection. A recent review suggest that Lassa fever poses an increased risk of mortality in pregnant women when compared to non-pregnant women (three times greater odds). [7] The review also showed high rates of foetal (61.5%) and neonatal losses (30.2%) suggesting that pregnant women, their foetuses and new-borns are particularly prone to adverse outcomes from Lassa fever. [7] Additionally, reports from West Africa suggest that Lassa fever is likely a major contributor to maternal mortality in endemic areas. [8, 9] Given that Lassa virus infection in pregnancy poses a significant risk of infection and adverse outcomes to the pregnant woman, and her foetus; pregnant women should be considered a ‘special group’ for interventions to prevent Lassa virus infection and disease. [7]

There are currently no licensed antivirals for the management of Lassa fever. [10] Ribavirin is used off-label for the management of Lassa fever. [10] However, the evidence justifying the use of ribavirin is poor, [11] and underscores the need for improved care and trials to obtain safety and efficacy data for therapeutics, [12] particularly in pregnancy. There are currently no vaccines for Lassa fever, however, amongst the viral haemorrhagic fevers, Lassa fever has one of the most advanced vaccine development platforms with six vaccines funded by The Coalition for Epidemic Preparedness Innovations (CEPI) which supports development of vaccines for emerging pathogens. [13] Most vaccine candidates are in preclinical trials, but there are currently three vaccines in Phase I human trials, Inovio’s DNA vaccine completed recruitment in 2022 for a Phase IB trial in Ghana; [14, 15] while IAVI’s vaccine and Emergent BioSolutions’s vaccine began Phase I First in human clinical trials in Liberia and Ghana respectively. [15] This underscores the need for further studies particularly clinical trials.

Inclusion of pregnant women in therapeutic trials would improve our understanding of the effects of various treatments in pregnancy. Similarly, inclusion of pregnant women in future vaccine trials would facilitate evaluation of vaccine efficacy and effectiveness in the context of pregnancy and provide evidence for vaccine program implementation, ensuring that pregnant women are not excluded from the benefits of vaccination. [12] However, both vaccine and therapeutic studies require evidence on the disease epidemiology.

Epidemiological evidence to support the design of vaccine or therapeutic trials is significantly limited with critical gaps in data on prevalence, infection incidence, risk groups, disease presentation, and antibody kinetics, and these gaps are even more acute for pregnant women. [7, 16] Evidence on prevalence and incidence facilitates the assessment of disease burden and the identification of risk factors for infection, which in turn support the development of treatment and control strategies and the planning and allocation of resources.

In the general population, over 80% of Lassa virus infections are undiagnosed or unreported, [5, 17] as such, the rates of Lassa virus infection (incidence estimates) inferred from clinically apparent cases alone would be a substantial underestimate. The best method for estimation of the Lassa fever burden would be through serological testing; that is, the prevalence of antibodies in the population of interest using assays that are highly sensitive and specific. [18] Data from West Africa have shown a high prevalence of Lassa virus antibodies (LASV IgG) in the general population with wide variation between and within countries, ranging from 8–52% in Sierra Leone, [5, 6] 21–58% in Nigeria, [19, 20] 4–55% in Guinea, [2, 5, 21] to 3–14% in Liberia. [22, 23] Annual seroconversion rates in the general population are estimated to range from 4.2–8.4% in Mali [24] to 5–22% in Sierra Leone. [6] Among pregnant women, one estimate of prevalence, obtained from a single study in Nigeria over a nine-year period, was 0.87% (44/5048). [9] This period prevalence was based solely on active cases or symptomatic Lassa fever pregnant women, and as such, is possibly a considerable underestimation of Lassa prevalence in pregnancy. It is likely that, similar to the general population, most Lassa virus infections in pregnancy are undiagnosed.

To the best of our knowledge, no previous studies have estimated Lassa seroprevalence, seroconversion or seroincidence in pregnancy. This study sets out to estimate the prevalence of prior LASV exposure (seroprevalence) and the incidence of new Lassa virus infections (seroconversion) in pregnant women and evaluate risk factors associated with seroconversion and seroprevalence for a better understanding of the epidemiology of Lassa fever in preparation for future Lassa vaccine and therapeutic trials.

## Methods

### Ethics statement

Ethical approval was obtained from the Oxford Tropical Research Ethics Committee (OxTREC) of the University of Oxford (OxTREC reference No.: 49–18), and the Irrua Specialist Teaching Hospital Research Ethics Committee (ISTH REC), Edo State, Nigeria (HREC Approval No.: NHREC/29/03/2017). Written informed consent was obtained from all participants.

### Study endpoints

To the best of our knowledge, no previous studies have estimated seroprevalence or seroconversion in pregnant women alone and as such we set it up as an exploratory study with no pre-defined hypothesis. Table 1 summarises the objectives of our study and the expected outcomes.

**Table 1:** Study objectives and outcomes measures.

	Objectives	Outcome measures
**Primary objectives**	To estimate the proportion of the pregnant population susceptible to Lassa fever in Irrua, Nigeria	Seroprevalence of LASV IgG antibodies in the study population
	To estimate the proportion of pregnant women seroconverting between first and second blood collection.	The incidence proportion of seroconversion (seroconversion risk)
**Secondary objectives**	To assess factors associated with maternal Lassa virus (LASV) seropositivity in pregnant women	Association between putative risk factors and IgG seropositivity (measured by odds ratio)
**Post-hoc**	To estimate the proportion of women seroreverting between first and second blood collection	Seroreversion risk

### Definition of endpoints

Further details on Lassa antibodies and the ELISA used for this study are found in the section on sample collection and laboratory analysis.

Seropositivity was defined based on cut-offs for LASV IgG antibody levels for the ELISA, as such LASV seropositivity was defined as samples with an antibody index value of 1.1 or above (≥1.1).

Seronegativity was defined as antibody index values of 0.9 or below (≤0.9). Susceptible pregnant women were defined as the proportion of pregnant women who were negative for IgG antibodies.

Seroconversion was defined as a participant who was seronegative for LASV IgG at baseline (index value ≤0.9), became seropositive at delivery (IgG index value ≥1.1), was in the study for at least 30 days, (that is the interval between the first maternal sample and the second maternal sample was ≥30 days) and had a change in the index value of four-fold or more.

A lower limit of 30 days was chosen because studies suggest that IgG has a mean time to first detection of 25.6 ± 3 days after symptom onset. [25] A four-fold rise in the index value was used because a four-fold increase in antibody titre is conventional for an antibody change defining infection and seroconversion. [26, 27] A fold change was calculated as the ratio of antibody concentration at delivery to that at enrolment.

Seroreversion was not an initial objective and was estimated post-hoc because of its relevance for disease burden estimates and preventative strategies. No standard definition exists for LASV IgG seroreversion, thus we chose to define seroreversion as a change from positive (index value≥1.1) to negative (index value ≤0.9) plus a four-fold decrease in concentration. [6]

### Study population and study procedures

The study was designed as a prospective hospital-based cohort, with pregnant women enrolled at the antenatal clinic and followed up at delivery to assess seroconversion rates. The inclusion criteria were pregnant women aged 15 years and above, attending antenatal clinics (ANC), and who were willing and able to give informed consent. Exclusion criteria comprised failure to give consent, withdrawal of consent, contraindication to venepuncture, absent or unavailable legally authorised representative, and patients for end-of-life care.

Participants were recruited between 12th February 2019 to 4th May 2019 from antenatal clinics (ANC) at Irrua Specialist Teaching Hospital (ISTH) and two of health centres in Edo, Southern Nigeria and followed up to delivery with the last participant delivered on December 20^th^, 2019. The health centres (Usugbenu primary centre and Eromosele medical centre) were randomly chosen from a list of facilities that frequently refer patients to ISTH and that were willing to participate in research studies. This period covered the known peak season for Lassa incidence in Edo state and in Nigeria which runs from December to May particularly January to March. [3, 28]

Evidence suggest that in developing countries while over 70% of pregnant women may attend ANC at least once, only 20–40% deliver in a health facility. [29, 30] Given this substantial risk of loss to follow up and coupled with the asymptomatic nature of Lassa fever; the sample size calculation was carried out based on a cross-sectional study design to detect seroprevalence at two time points (ANC and delivery). Evidence in the general population suggest that Lassa seroprevalence ranges from 21–58% in Nigeria [19, 20] taking a conservative prevalence of seropositivity of 30% and an absolute precision of 7.5% at a 95% confidence interval, the sample size was calculated at 144 (S1 File). However, demographic surveillance at Irrua Specialist Teaching Hospital (ISTH) shows that only 50–65% of pregnant women who attended ANC at ISTH deliver at ISTH. Thus, with an estimated loss to follow up of 40% between enrolment (ANC) and delivery, the sample size was increased to 240 pregnant women.

**Fig 1:**
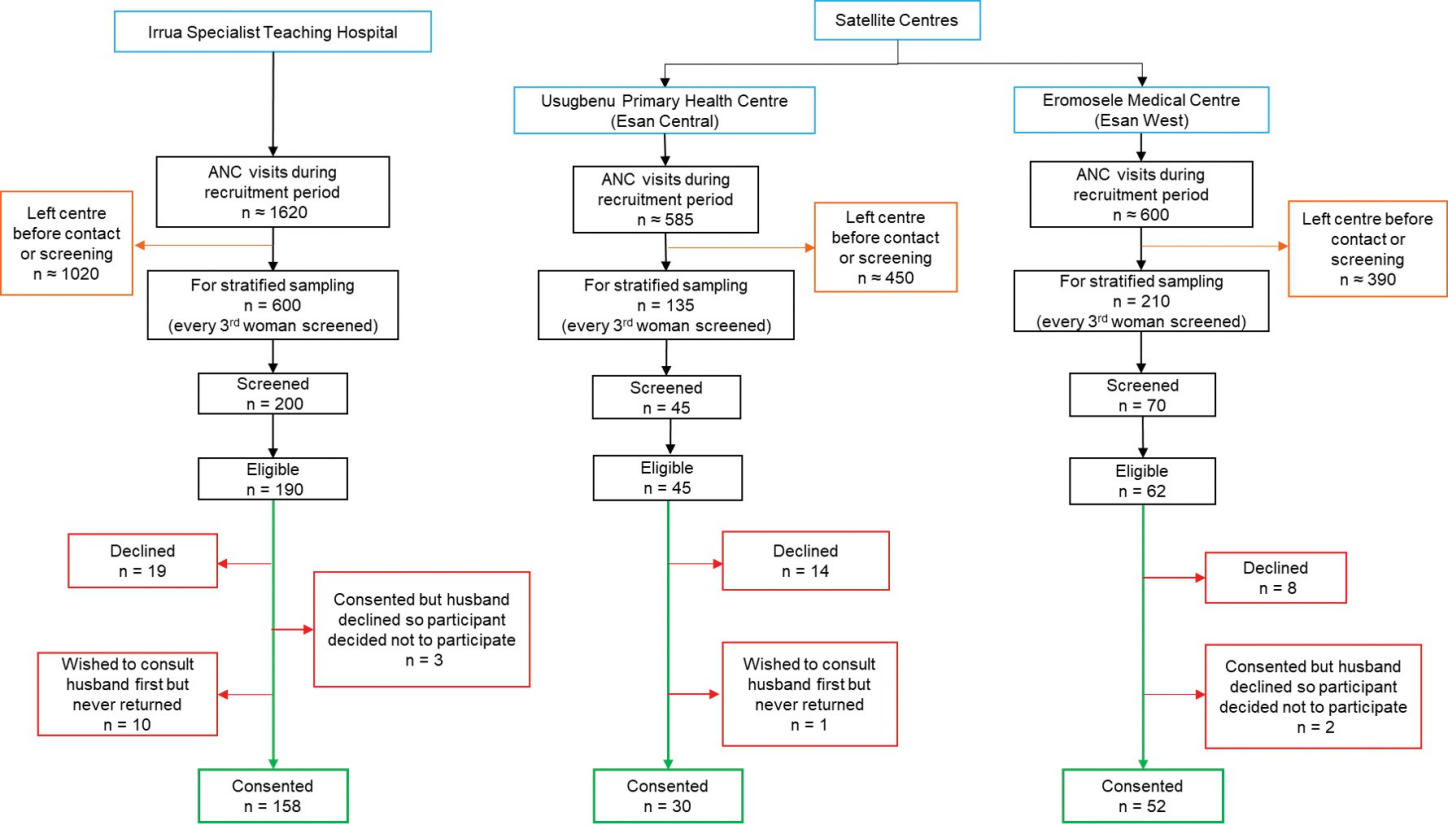
Flow diagram for participant recruitment. Note: Recruitment took place from 12^th^ February to 4^th^ May 2019. The number of possible participants who left before they were contacted is large because the study team had to divide their time between busy work schedules and the study.

At the ANC, a member of the study team (health care workers) introduced the study to all pregnant women attending the health education sessions. A stratified sampling technique was used where every third pregnant woman was screened for eligibility. Participants who were eligible and consented were enrolled in the study (S2 File). These participants were followed up at delivery. Fig 1 shows the flow diagram for enrolment.

A structured questionnaire was verbally administered in local languages (English and Pidgin) at enrolment and within 48 hours of delivery to collect clinical and demographic information. At enrolment we collected information on age, gestational age, residence in the 6months prior to enrolment, educational level, occupation, knowledge of Lassa fever risk factors and transmission, history of rodent exposure in the 6 months prior to enrolment, history of fever in the two weeks before enrolment, history of Lassa fever and other medical conditions such as diabetes, HIV, and hypertension, history of pregnancy related complications such as pregnancy induced hypertension (S3 File). A second questionnaire was completed within 48 hours of delivery to collect clinical data on residence between ANC and delivery, mode of delivery, birth weight of the neonate, sex of neonate, gestational age at birth, Apgar score at birth, fever between ANC and delivery, medical conditions between ANC and delivery, pregnancy related complications between ANC and delivery. (S4 File).

Data were later entered and managed using REDCap (Research Electronic Data Capture) electronic data capture tools. [31] The gestational age was estimated using fundal height and obstetric ultrasound at enrolment and using the Ballard Score [32] at birth. Clinical data on birthweights, malaria, HIV, Lassa fever, diabetes, hypertension, and other medical conditions were based on patient responses and verified using patient-held ANC books and/or patient hospital records. During training for data collection staff were told if patients reported illness to cross check in case notes (hospital records or ANC booklet); previous illness data was not further differentiated as self-reported, or laboratory confirmed illness. Gestational age below 37 weeks was defined as prematurity, and birthweight below 2.5Kg was defined as low birth weight. Rat exposure was retrospective and self-reported, here, the term rat is used to refer to small rodents found peridomestically; participants were asked about a history of eating rodents, rodents in homes, rodent burrows around the house and killing rodents found in homes in the 6 months prior to enrolment. We did not provide any advice on Lassa control as part of this study.

### Sample collection and laboratory analysis

Immune and pathophysiologic responses to Lassa virus are poorly understood. LASV-IgM rises by the second week of illness, [2, 25, 33, 34] with a mean time to first detection of 13.1 ± 0.9 days post-symptom onset. [25] Unlike in some viral infections, LASV IgM is not an appropriate marker for acute infection because LASV IgM can last over 12 months, [34] has been found in healthy controls, [34] may be falsely negative early in infection, and in endemic areas can lead to false positives due to immune responses to other common infections such as malaria. [35] As such, LASV IgM is possibly of ‘little or no diagnostic value’, particularly in endemic areas. [34, 35]

Studies suggest LASV IgG antibodies are produced in about the third week of illness [25] with a mean time to first detection of 25.6 ± 3days after symptom onset. [25] LASV-IgG concentration may remain constant for over 12months. [34] The presence of LASV IgG is usually suggestive of past infection. However, LASV IgM and IgG may rise simultaneously, [35] and LASV IgG has been detected in some patients within a few days of infection, [1, 33, 36] and in some cases, was detected earlier than IgM. [35] As such, recent infection and seroconversion may also be diagnosed by detection of LASV IgG particularly in follow-up samples. [35]

LASV has a high genetic diversity as such, there is currently no gold standard or reference assay for definitive laboratory diagnosis or serological testing of Lassa fever. [37, 38] The assay used in this study was one developed and previously used in this region showing a high specificity of 95–100%. [35]

Whole blood samples (2 – 5mL) were collected from mothers at enrolment and within 48 hours of delivery, and from the placental end of the umbilical cord immediately upon delivery. Samples were then centrifuged and aliquots (0.1–0.2mL) stored at –20°C until February 2020 when LASV IgG antibody ELISA (enzyme-linked immunosorbent assay) was performed using BLACKBOX LASV IgG ELISA (Diagnostics Development Laboratory, Bernhard-Nocht Institute for Tropical Medicine, Hamburg, Germany). [35] It is an immune-complex binding ELISA with antibody levels expressed as an index value (IV), calculated using optical density (OD) values. An index value of 1.1 or above (≥1.1) was considered positive for past or current infection, 0.9 or below (≤0.9) was negative, and values between 0.9 and 1.1 were equivocal. Serology was performed following the manufacturer’s instruction, which can be obtained freely from the European Virus Archive at https://www.european-virus-archive.com. All samples were stored on site at ISTH in Nigeria and while all contingencies were taken to ensure cold chain maintenance. In February 2020, prior to analysis, a fault in the backup generator resulted in our samples thawing, samples were refrozen but given that there is no data on the effect of freeze thaw cycles on Lassa antibodies, our findings need to be taken in light of this consideration.

### Statistical analysis

Data were analysed using R statistical software version 4.0.2. [39] Descriptive data analyses were performed to summarise the main characteristics of pregnant women. Summary statistics were presented as mean, median or frequencies depending on the variable. Non-parametric tests were used unless the Shapiro-Wilks test was not significant.

Overall seroprevalence was defined as pregnant women who were seropositive at any point during the study period. The numerator was pregnant women who were LASV IgG positive either at baseline or at delivery (Index value ≥1.1), and the denominator was the total number of pregnant women recruited into the study (N = 240). The 95% confidence intervals (CI) for the proportion was calculated using Wilson/Brown intervals for binomial proportions. [40]

Univariable logistic regression was performed to evaluate the association of different maternal characteristics with overall seropositivity, and these were reported as crude odds ratios (OR) with their corresponding 95% CI. Given that the overall seroprevalence included a varying number of visits (enrolment ± delivery), we performed an initial analysis to identify factors associated with seropositivity after controlling for the number of visits (baseline model). Factors that were remained marginally significant in the baseline model (P < 0.25) were included in the multivariable model. [41] Given past reports on a link between rodent exposure and Lassa fever, [42, 43] we calculated an attributable risk proportion for rodent exposure (S1 File).

Seroconversion risk was calculated as the proportion of pregnant women in the study population who seroconverted amongst the susceptible pregnant women (that is those who were negative for LASV IgG at baseline). The 95% CI for the seroconversion risk was calculated using Wilson/Brown intervals for binomial proportions.[40] Maternal factors were assessed for their association with seroconversion using the Fisher’s exact test, χ^2^ test and Mann-Whitney U test as appropriate, and a logistic regression was performed. Factors that were significant in the univariable model (*P*< 0.25) were included in the multivariable model. [41] When performing a conventional regression with small sample sizes or rare events, there is a risk of quasi or complete separation of the model, and a likelihood of underestimating the true effect size; as such we used the Firth or penalised maximum likelihood regression which allows for calculation of finite and consistent estimates in the regression parameters. [44, 45]

We estimated seroreversion risk post-hoc. Seroreversion was calculated as the number of pregnant women who were LASV IgG positive (index value ≥1.1) at baseline but were seronegative (index value ≤0.9) at delivery.

For all regressions, there were no imputations for missing data; consequently, denominators vary by response. Analysis was conducted in three steps. First, we performed a univariate analysis to explore variables for inclusion in a multivariable model. A P value of <0.25 was set as cut-off, that is the *“P to remove”*. [46] Next, we performed a multivariable regression where a two-sided P-value of ≤ 0.05 was considered statistically significant. However, to account for multiple comparisons and given that the study was exploratory and does not have a pre-defined hypothesis, the Bonferroni correction was applied. [47] To prevent loss of information and the risk of increasing type-1 error, continuous variables were not categorised in the regression models, [48, 49] thus in all regressions, age, gestational age, and parity were not categorised, when evaluating their correlation with seropositivity or seroconversion.

Collinear terms for the study were age and parity; gestational age at birth and birth weight; and fever during pregnancy and malaria during pregnancy. Malaria, age and gestational age at birth are more biologically relevant for changes in immune responses (antibody changes). [35, 50–52] As such, if both collinear terms were significant in the univariable model, then only age, malaria, or gestational age at birth were included in the multivariable model.

## Results

The study results were reported following the Reporting Of SeroEpidemiologic Studies for Influenza (ROSES–I) statement, [53] which is easily adaptable to other infections.

### Baseline characteristics

A total of 240 women were enrolled into the study. Most participants were recruited during the known peak incidence period of Lassa fever in Nigeria which runs from December to May with the highest peaks observed between January and March. [3].

**Fig 2:**
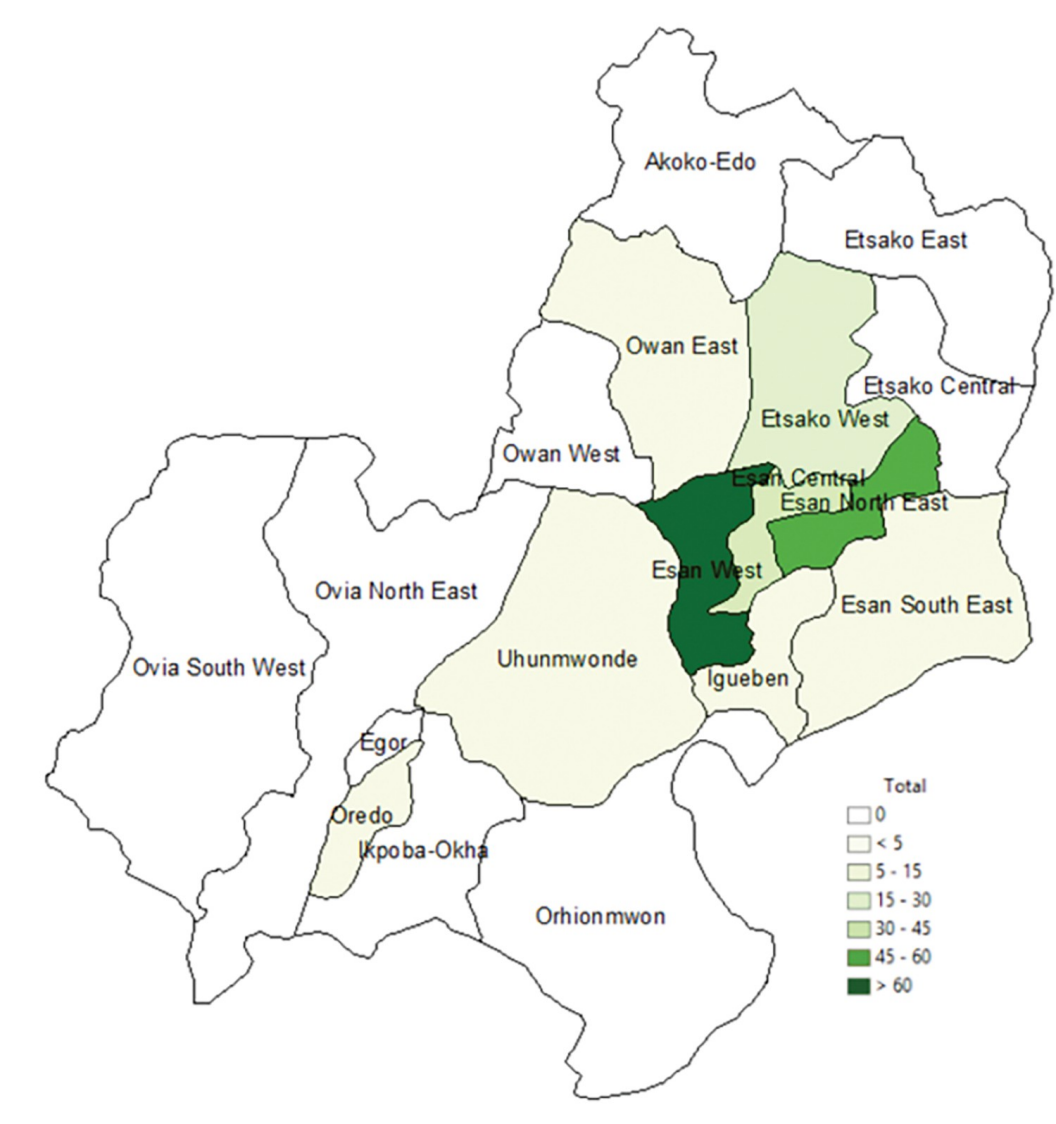
Map of Edo state indicating where participants lived six months before enrolment and throughout the study. Shapefiles for Edo LGA obtained from Taiwo[54] figshare repository under creative commons CC BY 4.0.

The mean age of the study participants was 31.6 ± 5.0 years [range: 20–46 years] and pregnant women above 30 constituted 55.8% of the total study population. Most participants (79.1%) had at least one previous pregnancy (range: 2–8) with the majority in their second trimester of pregnancy (51.7%) and a median gestational age of 26 weeks [interquartile range (IQR): 18–31 weeks]. All participants had some form of formal education, 69.6% had completed secondary school and 54.2% lived in rural areas. Knowledge of Lassa fever risk factors and transmission was generally poor (87.5%). One hundred participants (41.7%) were employed in the informal sector (mainly as traders, tailors, hairdressers, and farmers). Most participants were from Esan West secondary administrative district or local government area (LGA), Fig 2. Details of the study participants’ characteristics at baseline are provided in Table 2.

**Table 2:** Baseline characteristics of pregnant women.

Characteristics	n	%
**Total**	240	
**Age (years)**–Mean age ± SD [range]	31·57 ± 5·01 [20–46]	
≤ 25	33	13·8
26–30	73	30·4
31–35	90	37·5
36–40	37	15·4
> 40	7	2·9
**Gestational age**^**a**^ (weeks)–Median [IQR]	26 [18–31]	
≤ 13	34	14·5
14–27	121	51·7
> 28	79	33·8
Lives in a rural area	130	54·2
**Educational level** ^**b**^		
Primary	11	4·6
Secondary	62	25·8
Completed Secondary (Post-secondary)	167	69·6
**Occupation**		
Student	15	6·3
Housewife	39	16·3
Health Professional	25	10·4
Informal Sector	100	41·7
Formal Sector	61	25·4
**Parity**		
0	50	20·8
1–2	98	40·8
≥ 3	92	38·3
Poor knowledge of Lassa fever transmission and risk factors	210	87·5
Positive history of exposure to rodents in the last 6 months	191	79·6
Possible exposure to Lassa fever infected patients in last 6 months	14	5·8
Positive history of fever during pregnancy	14	5·8
Positive history of Lassa fever	5	2·1
Positive history of malaria during pregnancy	64	26·8
Hypertension	5	2·1
Pregnancy-induced hypertension	2	0·8
Diabetes mellitus	6	2·5
Gestational diabetes	3	1·3
HIV–infected	14	5·8

The table describes the characteristics of study participants at baseline.

**Note:** DM- diabetes mellitus; HIV- human immunodeficiency virus; IQR- interquartile range; n- number of pregnant women recruited into the study; SD- standard deviation.

^a^ Data missing for 6 women.

^b^ All participants had some form of formal education.

### Lassa IgG seroprevalence

The overall seroprevalence (at baseline or delivery) during the study period was 49.6% [95% CI: 43.3–55.9%], with only 1.3% (3/240) of the seropositive participants reporting a prior history of a Lassa fever diagnosis. Serostatus was available at baseline for all participants and was available at delivery for 172 participants. The seroprevalence at enrolment was 40.4% [95% CI: 34.0–46.3%], and at delivery was 45.7% [95% CI: 38.4–53.1%]. This shows an increase from baseline; however, this increase was not statistically significant (McNemar’s test; p = 0.052). The mean age among seropositive mothers was 31.8 ± 5.4 years [range: 20–45] and was not significantly different from that of seronegative mothers 31.3 ± 4.6 [range: 22–46] (t-test; p = 0.345). The median gestational age at recruitment was 24 weeks [IQR: 18–30] among seropositive mothers and 26 weeks [IQR: 21–32] among seronegative women (Mann–Whitney p = 0.276). The median time spent in the study was 107 days [Interquartile range (IQR): 55–154 days].

In a multivariable logistic regression, a self-reported prior history of Lassa fever was not significantly associated with seropositivity, and residence in a rural area was not significantly associated with seropositivity (OR, 1.42 [95% CI: 0.89–2.68], p = 0.126). Before accounting for multiple comparisons, a positive history of rodent exposure (OR, 2.32 [95% CI: 1.18–4.70, p = 0.015]) and HIV infection (OR, 5.16 [95% CI: 1.22–36.4, p = 0.046]) were significantly associated with LASV IgG seropositivity, Table 3. However, when accounting for multiple comparisons using the Bonferroni correction, none of the variables remained statistically significant.

**Table 3:** Factors associated with Lassa maternal IgG seropositivity at baseline or delivery.

Factor	N	n	OR crude [95% CI]	P crude	OR adjusted ^a^ [95% CI]	P adjusted^a^
**Age (years)**	240	119	1·02 [0·98–1·08]	0·294	—	—
**GA at enrolment**^**b**^ **(weeks)**	234	116	0·98 [0·95–1·02]	0·259	—	—
**Parity**	240	119	1·15 [1·01–1·32]	0·040	1·13 [0·96–1·32]	0·128
**Lives in a rural area**						
No	110	62	1·66 [1·00–2·79]	0·050	1·42 [0·89–2·68]	0·126
Yes	130	57	Reference		Reference	
**Educational level**						
Post-secondary	167	77	Reference		Reference	
No Post-secondary	73	42	1·67 [0·95–2·98]	0·075	1·25 [0·66–2·40]	0·397
**Occupation**						
Student	15	8	Reference			
Housewife	39	20	1·20 [0·36–4·05]		—	—
Health professional	25	8	0·53 [0·14–2·01]	0·651^c^	—	—
Informal Sector	100	54	1·37 [0·45–4·18]		—	—
Formal Sector	61	29	1·09 [0·35–3·49]		—	—
**Knowledge of LF**						
Good	30	11	Reference		Reference	
Poor	210	108	1·83 [0·84–4·17]	0·132	1·87 [0·81–4·48]	0·146
**Exposure to rodents**						
No	49	17	Reference		Reference	
Yes	191	102	2·14 [1·13–4·20]	0·022	2·32 [1·18–4·70]	0·015
**Possible exposure to LF patients**						
Unlikely	226	114	Reference		—	—
Likely	14	5	0·53 [0·16–1·58]	0·269	—	—
**Fever during pregnancy**						
No	198	94	Reference		—	—
Yes	42	25	1·60 [0·83–3·20]	0·174	—	—
**Positive history of Lassa fever**						
No	235	116	Reference		—	—
Yes	5	3	1·52 [0·25–11·7]	0·651	—	—
**Malaria during pregnancy**						
No	143	65	Reference		Reference	
Yes	97	54	1·52 [0·90–2·55]	0·118	1·60 [0·91–2·85]	0·103
**Hypertension**						
No	235	116	Reference		—	—
Yes	5	3	1·46 [0·24–11·3]	0·680	—	—
**Pregnancy-induced hypertension**						
No	232	114	Reference		—	—
Yes	8	5	1·76 [0·42–8·76]	0·447	—	—
**Diabetes Mellitus** ^**d**^						
No	231	117	Reference		Reference	
Yes	9	2	0·28 [0·04–1·19]	0·119	0·30 [0·02–1·01]	0·104
**HIV–infected**						
No	226	107	Reference		Reference	
Yes	14	12	7·01 [1·84–45·9]	0·012	5·16 [1·22–36·4]	0·046

**Note: For statistical significance, Bonferroni p <0·005**; CI- confidence interval; GA- gestational age; LF- Lassa fever; n- number of seropositive women; N- total number of women who were enrolled into the study (240); OR- odds ratio.

^a^ Adjusted for factors which were marginally significant in the univariate regression (crude p<0·25), except collinear terms.

^b^ Data missing for 6 women.

^c^ Overall P value.

^d^ None of the women with gestational diabetes were seropositive thus diabetes is evaluated as one group to prevent separation in the model.

The attributable risk proportion due to rodent exposure was estimated to be 35.03%. Rodent exposure was self-reported and included rodents in homes, rodent burrows around the house or killing rodents found in homes. When evaluating factors at baseline, HIV status was the only variable which remained significantly associated with seropositivity in the multivariable regression, and when we accounted for multiple comparisons, none of the variables were significantly associated with baseline seropositivity. S1 Table summarises the risk factors for seropositivity at baseline. At baseline, two pregnant women had equivocal results and were excluded from the baseline seroprevalence analysis. There were no equivocal results at delivery. Of the two patients who had equivocal results at baseline, we found that at delivery one was positive, and the other was negative.

### Lassa IgG seroconversion and seroreversion

Of the 240 participants who were recruited, 67 (27.9%) were lost-to-follow-up (LTFU). The difference between various maternal characteristics amongst those LTFU and those retained was not statistically significant for most variables. The only maternal characteristic affected significantly by LTFU was the educational level, where 43.6% of women with secondary education were LTFU compared to 27.3% with primary education and 22.2% with post-secondary education (p = 0.007), (S2 Table). This resulted in those with post-secondary education being over-represented in this cohort of pregnant women.

One hundred and seventy–three pregnant women participated at both baseline and delivery, of these, 106 were seronegative at baseline (Lassa naïve/susceptible). Amongst those who were susceptible at baseline, 22 satisfied the criteria for seroconversion, with an estimated seroconversion risk of 20.8% [95% CI: 14.1–29.4%] amongst pregnant women. Amongst the women who seroconverted, the median time spent in the study was 144 days [IQR: 125.5–183.0 days].

Overall, the mean age for LASV IgG seroconversion was 32.9 ± 5.9years [range: 21–41] but was not significantly different from those who remained at risk (Mann-Whitney U p = 0.099). At baseline, five women had self-reported history of Lassa fever, three of these women were seronegative at baseline and had samples at both baseline and delivery. Of these, one satisfied the criteria for seroconversion. There was a positive history of fever during pregnancy in six of the women who seroconverted, Table 4.

In a multivariable regression model (Table 4), a positive history of rodent exposure was associated with increased odds of seroconversion (OR, 11.77 [1.46–1523], p = 0.014). However, when accounting for multiple comparisons with the Bonferroni correction, none of the variables remained statistically significant.

**Table 4:** Factors associated with Lassa IgG seroconversion during pregnancy.

Factor	N	n	χ^2^ P value^a^	OR crude [95% CI]	P crude	OR adjusted^b^ [95% CI]	P adjusted^b^
**Total**	106	22					
**Age (years)**	106	22	0·099	1·06 [0·97–1·17]	0·211	1·03 [0·94–1·14]	0·557
**Parity**	106	22	0·318	1·20 [0·90–1·59]	0·204	—	—
**Lives in a rural area**							
No	42	12	0·142	2·13 [0·84–5·50]	0·112	2·34 [0·86–6·77]	0·097
Yes	64	10		Reference		Reference	
**Educational level**							
Post-secondary	83	14		Reference		Reference	
No post-secondary	23	8	0·081	2·63 [0·94–7·19]	0·066	2·65 [0·82–8·49]	0·101
**Occupation**							
Student	8	3		Reference		—	—
Housewife	15	2		0·29 [0·04–1·92]		—	—
HCP	17	4	0·713	0·52 [0·09–3·04]	0·724^c^	—	—
Informal Sector	34	6		0·36 [0·07–1·88]		—	—
Formal Sector	32	7		0·46 [0·09–2·39]		—	—
**Knowledge of LF risk & transmission**							
Good	17	2		Reference		—	—
Poor	89	20	0·515	1·83 [0·51–9·79]	0·381	—	—
**Exposure to rodents**							
No	22	0		Reference		Reference	
Yes	83	22	0·006	16·19 [2·07–2088]	0·003	11·77 [1·46–1523]	0·014
**Possible exposure to LF patients**							
Unlikely	97	20		Reference		—	—
Likely	9	2	1·00	1·26 [0·22–5·18]	0·768	—	—
**Fever during pregnancy**							
No	87	16		Reference		Reference	
Yes	19	6	0·218	2·08 [0·68–6·02]	0·192	1·80 [0·48–6·26]	0·368
**History of Lassa fever**							
No	103	21		Reference		—	—
Yes	3	1	0·506	2·30[0·20–18·24]	0·451	—	—
**Malaria during pregnancy**							
No	67	13		Reference		—	—
Yes	39	9	0·804	1·25 [0·48–3·21]	0·635	—	—
**Hypertension**							
No	104	21		Reference		—	—
Yes	2	1	0.881	3·88 [0·30–49·7]	0·265	—	—
**Pregnancy induced hypertension**							
No	103	20		Reference		Reference	
Yes	3	2	0·205	6·79 [0·86–77·2]	0·069	8·18 [0·95–101]	0·055
**Diabetes mellitus**							
No	104	22		Reference		—	—
Yes	2	0	1.00	0·73 [0·005–9·44]	0·838	—	—
**Gestational Diabetes**							
No	102	22		Reference		—	—
Yes	4	0	0·578	0·39 [0·003–3·95]	0·493	—	—
**HIV–infected**							
No	105	21		Reference		Reference	
Yes	1	1	0·207	11·79 [0·61–1745]	0·101	4·03 [0·17–654]	0·397

**Note: For statistical significance, Bonferroni p<0.008;** CI- confidence interval; GA- gestational age; HCP- health professionals; LF- Lassa fever; n- number of women who seroconverted; N- total number of women who were seronegative at baseline (enrolment); OR- odds ratio; χ^2^- Chi squared.

^a^ Fisher’s Exact p value or χ^2^ p value as appropriate.

^b^ Adjusted for factors which were marginally significant in the univariate regression (crude p<0·25), except collinear terms.

^c^ Overall P value.

Seroreversion was not one of the initial objectives of this study. However, of the 67 pregnant women who were seropositive at baseline and who returned at labour/delivery, nine seroreverted (13.4% [95% CI: 7.2–23.6%]), that is had a LASV IgG index value of 0.9 or below (≤0.9) at labour/delivery and this decrease was over four-fold for all nine sample pairs. Amongst the women who seroreverted, the median time spent in the study was 163 days [IQR: 124.8–182.8 days].

## Discussion

The study shows a high prevalence of prior LASV IgG antibodies (49.6%) in pregnant women attending antenatal clinics in this hyperendemic region (Central Edo State, Nigeria). This suggests a susceptibility of 50% in pregnant women during the study period, which is higher than susceptibility reported in the general population in this hyperendemic region (42%). [19] The recorded increase in prevalence seen between enrolment (40%) and delivery (45.7%) may be explained by the fact that 60–85% of the delivery data from our cohort was recorded after the yearly Lassa peak period in Nigeria. [3, 28] Over the last couple of years, data from ISTH and the region suggest an increasing trend in occurrence of Lassa fever. [3, 55] For instance, in 2018 the total number of cases was 218 and increased to 289 in 2019 and 386 in 2020, compared to 112 in 2017 which suggest that our data was collected in a year where there was an increase in Lassa incidence. [56] Our findings therefore need to be taken in light of this consideration.

Our study had two time points, as such, we could not estimate the exact time at which seroconversion occurred or identify the time at which women were lost-to-follow-up. As a result, we could not estimate the incidence rate but rather report on the incidence risk (seroconversion risk). The seroconversion risk in this cohort was 20.8%, which is much higher than seroconversion observed in the general population in the non-endemic region of Mali (4–8%)[24] but similar to that reported in other hyperendemic areas such as in Sierra Leone where seroconversion was reported to be between 5–22%. [6] It is unlikely that all of these are new incidences of Lassa fever, and supports the view that reinfection may occur and results in a mild form of illness. [6] Nevertheless, the findings underscore the need for larger prospective studies to understand the clinical course of LASV infection in pregnancy and the need for preventative and therapeutic solutions tailored to pregnant women.

Our data suggest a substantial seroreversion risk during the study period (13.4%), which is much higher than that estimated in the general population in Sierra Leone (6.4%). [6]. The presence of seroreversion has an impact on the estimation of the burden of Lassa fever in this region, particularly, the estimation of susceptibility in pregnancy and suggests that the prevalence obtained in this, and other cohorts are an underestimate of the actual proportion of women of childbearing age who present at pregnancy with prior LASV exposure. This is relevant because the impact of a vaccination programme depends in part on the risk of exposure, the susceptibility of the exposed population, and the probability of adverse outcomes once infected. Further studies are needed to provide a better understanding of the risk of seroreversion and the susceptibility of seroreverted pregnant women to LASV reinfection during pregnancy. Additionally, the occurrence of both seroconversion and seroreversion in this cohort suggests that these parameters would need to be considered for the development of Lassa vaccine efficacy, effectiveness, and utility models.

An alternative explanation for the observed seroconversion and seroreversion risk is potentially the cross-reactivity of the assay with other antigens, [57] which may result in false negatives or false positives. Similarly, lipaemia in the samples (Price C & Newman D 1997 ref. in Tate J et al. [57]), as well as repeated freeze-thaw cycles, [58] may result in false positives or false negatives and support the need for further studies. An IgG neutralisation test may have facilitated confirmation of seroconversion and seroreversion. [59] and would be worth exploring in another study.

The relatively high effect sizes for the association of rodent exposure to seroprevalence [OR, 2.32] and seroconversion [OR, 11.77], suggests that similar to the general population, pregnant women were more likely to get Lassa infection from zoonotic spill over rather than from human exposures. [42, 43] With an attributable proportion of 35.0%, the data suggest that infections might be preventable by avoiding rodent exposure and conditions which facilitate infestation and the risk of human-rodent contact. Ecological studies from Nigeria suggest that while in most areas the main reservoir for Lassa fever (*Mastomys natalensis*) was found indoors, in some urban areas in Edo state, these small mammals were mainly found outdoors. [60] As such, given that our study did not categorise the type of rodents or quantify exposure, future studies particularly One Health studies. [17, 61] are needed to provide a better understanding of the avenues of human-rodent interaction in pregnancy to support development of effective control strategies. [17, 62–64] In the interim, advocating public health measures for rodent control such as proper sanitation and hygiene, improved housing and health education could support Lassa fever control efforts. [64, 65].

Contrary to previous studies, [66, 67] we found there was no significant difference between seropositivity amongst pregnant women living in rural areas compared to those in urban areas. There was also no association found between LASV seropositivity and educational level, unlike in previous studies in the general population in Edo state.[68] Our findings can probably be explained by the fact that post-secondary educated participants were over-represented in our cohort. However, community-based studies in this region have also reported high levels of formal education, [69] signifying that this region may have higher literacy levels. Despite the over-representation of post-secondary educated participants in this cohort, knowledge on Lassa fever transmission and risk factors was generally poor (87.5%). Further studies would be useful to understand the interrelation between education and seropositivity and identify knowledge gaps and behaviours that impede or promote Lassa control particularly in pregnant women.

Our cohort had a mean age of 31.6±5 years which is higher than reports on the national average in Nigeria, which in 2018 was 26 years. [70] The relatively high age of women in our cohort, may be explained by the over-representation of post-secondary educated (55.8%) and multiparous women (71.9%) in our cohort. Additionally, our cohort was recruited from health facilities and studies suggest that in Nigeria, younger women (<25years) are less likely to attend or deliver in health facilities. [71] Demographic surveillance from Nigeria suggest that adults between 21–40 years were the age group most affected by Lassa fever in 2019, and may account for the high seroprevalence (49.6%) observed in our cohort. [72] Further community based studies with a pregnant cohort may provide a better understanding of the effect of age on Lassa seropositivity.

We recorded an association between HIV infection and LASV IgG seropositivity. While these associations were not statistically significant, the effect sizes were high and warrant further inquiry particularly given the negative consequences of these diseases in the pregnant woman and her foetus. [7] It is also possible that any correlation between Lassa fever and HIV may simply reflect their relationship to socioeconomic disparities, given that both Lassa fever [5, 68] and HIV [73] are considered diseases of poverty; although evidence in HIV is conflicting and context-specific.

### Limitations

The use of a cohort design brings the added risk of loss to follow up (LTFU); we addressed this by adjusting our sample size to accommodate LTFU at rates equivalent to the average differences generally observed between ANC and institutional delivery at ISTH. The actual LTFU was lower than we had accounted for in the sample size.

Most of the pregnant women enrolled in the study were in their second trimester of pregnancy. This is unsurprising because pregnant women in Sub-Saharan Africa tend to attend ANC after the first trimester. [74–77] However, it did not affect the estimate for seroconversion risk because the definition for seroconversion included a time frame allowing only women with over 30 days in the study to be evaluated as seroconverts.

While a seroconversion risk of 20.8% is relatively high, in terms of absolute numbers, the number of seroconversion events was small and given the extremely wide confidence intervals, the estimates for potential risk factors may not be precise, however, the study highlights potential areas for further inquiry and provides a baseline on seroconversion in pregnancy which can facilitate design of larger prospective studies.

## Conclusion

The study found a prevalence of 49.6% for Lassa IgG antibodies in pregnant women in Edo State, Nigeria with an estimated attributable risk proportion of 35% for rodent exposure. However, the evidence on rodent exposure in our study was subjective and further studies are needed to provide a better understanding of the avenues of human-rodent interaction and Lassa control in pregnancy. In the interim, rodent control measures would be beneficial. While the evidence is not conclusive, our study provides a baseline for seroconversion in pregnancy to facilitate future prospective studies. Moreover, the estimated seroconversion risk of 20.8% is similar to that observed in the general population in hyperendemic areas, and while most of these seroconversions may not be new infections, given the high risk of adverse outcomes in pregnancy, our study supports the need for preventative and therapeutic options against Lassa fever in pregnancy. The occurrence of seroreversion suggests that the prevalence obtained in this, and other cohorts may be an underestimate of the actual proportion of women of childbearing age who present at pregnancy with prior Lassa exposure. Additionally, the occurrence of both seroconversion and seroreversion in this cohort suggests that these parameters would need to be considered for the development of Lassa vaccine efficacy, effectiveness, and utility models. This study provides valuable information for estimation of the epidemiologic disease burden of Lassa fever, which contributes to an estimation of disease impact in the short term and long term, and as such, allows for planning and allocation of resources, and facilitates monitoring and evaluation of disease control policies.

## Supporting information

S1 File Sample size calculation and attributable risk proportion. (PDF)

S2 File Guidance for obtaining informed consent. (PDF)

S3 File Enrolment or baseline questionnaire. (PDF)

S4 File Delivery questionnaire. (PDF)

S1 Table Factors associated with Lassa maternal IgG seropositivity at baseline. (DOCX)

S2 Table Characteristics of participants lost-to-follow-up (LTFU) & an estimation of the effects of LTFU. (DOCX)
