# Supplementary material for: Seroepidemiology of Lassa virus in pregnant women in Southern Nigeria: A prospective hospital-based cohort study
Source: PLoS Negl Trop Dis. 2023 May 22;17(5):e0011354. doi: 10.1371/journal.pntd.0011354 (PMC10237645; doi:10.1371/journal.pntd.0011354)
Supplement: S1 File — (PDF) [file pntd.0011354.s001.pdf]

S1 File: Methods

Sample size calculation

The sample size formula was based on:

$$N = \frac{Z^2 p(1-p)}{e^2}$$ (1, 2)

Where N is the sample size, ρ is the prevalence, e is the absolute value of precision, and Z is the critical value from the standard normal distribution corresponding to the desired confidence level and is 1.96 for a confidence level of 95%.

Attributable risk proportion

The attributable risk fraction or proportion (ARP), defined as the proportion or percentage of Lassa fever cases which could be attributed to rodent exposure and which are (potentially) preventable by elimination of the risk of rodent exposure,(3, 4) was calculated as:

$$ARP = \frac{Pe - Pu}{Pe} \times 100$$ (3)

Where Pe is the proportion of pregnant women with a positive history of rodent exposure who were LASV IgG seropositive and Pu is the proportion of pregnant women without a positive history of rodent exposure who were LASV IgG seropositive.

References

1. Lwanga SK, Lemeshow S, World Health O. Sample size determination in health studies : a practical manual / S. K. Lwanga and S. Lemeshow. Geneva: World Health Organization; 1991.
2. Daniel W.W. Biostatistics: A Foundation for Analysis in the Health Sciences. 7th edition ed. New York: John Wiley & Sons; 1999.
3. Porta M. A dictionary of epidemiology. 6th ed. New York: Oxford University Press; 2014.
4. Gefeller O. An Annotated Bibliography on the Attributable Risk. Biometrical Journal. 1992 1992/01/01;34(8):1007-12.
