# Supplementary material for: Seroepidemiology of Lassa virus in pregnant women in Southern Nigeria: A prospective hospital-based cohort study
Source: PLoS Negl Trop Dis. 2023 May 22;17(5):e0011354. doi: 10.1371/journal.pntd.0011354 (PMC10237645; doi:10.1371/journal.pntd.0011354)
Supplement: S2 File — (PDF) [file pntd.0011354.s002.pdf]

**SOP Ref No** **Lassa virus in Pregnancy study_SOP_03**

**SOP title** **Gaining Consent**

**Version** **1.0**

**Date issued** **13th December 2018**

**Valid until** **13th December 2020 (maximum of 2 years)**

**Author(s)** **Name:** **Delphine Kayem**

**Date:** **13th December 2018**

**Modification history**

| Version No | Date | Author(s) | Date reissued to previous recipients |
|---|---|---|---|
| 1.0 | | Delphine Kayem | |
| | | | |
| | | | |
| | | | |
| | | | |
| | | | |

# Standard Operating Procedure

## 1 Purpose

The purpose of this standard operating procedure (SOP) is to ensure that all pregnant women (or their representatives) who are invited to participate in the Lassa virus in pregnancy study fully understand the risks and benefits of the study, what being in the study will involve, and that informed consent is recorded.

## 2 Scope

This SOP details how to perform, record and store informed consent for participation in the study for adults (aged 18years or over) and children (aged 15-18years), who meet the eligibility criteria.

## 3 Responsibility

Study staff will be responsible for ensuring pregnant women, guardians and/or other family members (as appropriate) are fully informed about the study and that informed consent is given freely. Study staff will also be responsible for ensuring informed consent is documented and filed according to this SOP. The study coordinator will be responsible for delegating study staff to obtain informed consent and ensuring they are listed on the delegation log.

## 4 Procedure

4.1 For full details on eligibility criteria, see Screening and Enrolment SOP01. For the purposes of taking informed consent, it is important to determine whether pregnant women are eligible to take part in the study. All these pregnant women will need to be added onto the participant Screening log F01. Pregnant women will be eligible to take part in the study if:

- Pregnant women attending antenatal clinic (ANC)
- Pregnant women who are willing and able to give informed consent for participation in the study
- Aged 15years or over (≥15years)

4.2 **The person to be asked for consent:**

- For adults that is women 18 years or more; the pregnant woman herself should be asked for consent unless she is deemed too unwell to give informed consent. If she is medically unable to give consent, then the clinical or treating staff will identify a legally authorised representative (LAR) to provide proxy consent. If there is no LAR available, then the participant should not be enrolled in the study. If the participant's health improves at any point she should be asked for her consent and this recorded.
- For children (pregnant women between 15 and 18 years of age), study staff should seek assent from the child and consent from the legally authorised representative (LAR) such as parent, legal guardian or close family member.

- Pregnant women/parents/guardians/representatives who do not wish to decide immediately or who have declined for themselves or their child to take part in this study may decide they wish to take part or that their child should take part up in this study at a later date. However this decision will need to take place before the end of the enrolment period that is 28/04/2019. After this time, they will not be eligible for entry into the study as recruitment is planned for completion on 28/04/2019.
- For the purposes of consent, the assessor must be satisfied that a n adult pregnant woman who is mentally fit can do all of the following:
  - Understand information given to them
  - Retain that information long enough to be able to make the decision
  - Assess the information available to make the decision
  - Communicate their decision.

**Consent must be taken by a member of study staff who has been given the authority to do so on the Delegation of Duties log.**

1. Pregnant women may be informed about the study and receive an explanation of the consent procedure by communicating verbally.
2. Ascertain the most appropriate language in which to discuss the study (English or a local language) and whether the participant is able to read English.
   - If the participant is unable to read English, ensure a witness is present before discussing the study further. The witness can be a member of Hospital/ clinical staff who is not directly involved in the study or the member of Hospital/clinical staff seconded to the study for this purpose.
3. Give a copy of the appropriate Participant Information Sheet (PIS) and Informed Consent Form (ICF), to the participant if she can read English.
   - Ensure the participant is given the correct consent form.
   - There is one Participant information Sheet, one Informed Consent Form and one Assent form: PIS_Lassa virus in pregnancy study_ v.1.1, Consent_Lassa virus in pregnancy study_ v.1.1, and Assent_Lassa virus in pregnancy study_ v.1.1.
4. Discuss the Participant Information Sheet and Informed Consent Form or Assent form with the participant.
5. Give the participant the chance to ask questions about the study.
6. Ask the participant whether she agrees to take part in the study and to each statement on the Informed Consent Form.
   - In the case of children (pregnant women below 18years of age), ask the child whether she agrees to take part in the study and to each statement of assent on the Assent form. (Remember for the child you also need to get consent from the LAR (parent, legal guardian or close family member).
7. Using the appropriate PIS/ICF/Assent form (check if it has the correct version number), record consent/assent for those who agree to take part in the study.
   - For participants who are able to read English, the person giving consent must sign and date (DD/MM/YYYY i.e. 01/01/2019) the consent form.
   - If the participant is unable to read English, the witness must sign and date (DD/MM/YYYY i.e. 01/01/2019) the consent form and the participant's thumbprint stamped onto consent form.

8. The study staff must countersign and date (DD/MM/YYYY i.e. 01/01/2019) the consent form in the appropriate box.
9. When the Informed Consent Form has been signed by relevant parties, submit all copies of the paper consent forms to the study co-ordinator.
10. Make sure you give the participant a copy of her consent/assent form.
    - If the participant is going to be admitted to hospital immediately, please leave a copy in the patient file (clinical notes) with an instruction for this to be given to the participant at discharge.
    - If the participant is admitted, make a note on the copy you give the study coordinator so he can follow up to ensure the participant has received her consent/assent form on discharge.
11. Add the pregnant woman's details to the pregnant woman Identification Log (Identification log_F02), Enrolment log_F06 and Informed Consent log_F03 kept in the Lassa virus in Pregnancy Master File on site.
12. For participants who are admitted, write in the pregnant woman's clinical notes to state that the pregnant woman met eligibility criteria and provided informed consent to participate in the study. Sign and date the notes entry was made. Please document clearly if proxy consent was needed with the names of the persons involved.
13. Allocate the pregnant woman a unique study number (participant code) by choosing a sticker from the roll provided. Place the sticker onto the patient antenatal clinic card, the patient file and another on to the paper questionnaire. The code should look like this

LVP-OX001

14. Record the participant code (by hand) on the participant Identification Log and the Enrolment Log.
15. If an excluded pregnant woman subsequently becomes eligible, they may still be consented to take part, as long as this occurs before 28/04/2019, the projected end of enrolment date.
16. For any woman who decides she wishes to consult her family before making a decision, or for women who refuse to participate,
    - Please write their screening number on the white cards provided.
    - Give the participant the white card
    - Explain that if they change their minds and decide to participate, they can return to the centre before 28/04/2019 with the white card
    - When she returns, write down her screening number with an asterix (*) beside it on the participant informed consent log.
    - Follow the usual consent and enrolment procedures thereafter.

## 5 Associated Documents

- SOPs:
    - Lassa virus in pregnancy study_Screening and recruitment_SOP_02
- Forms:
    - Lassa virus in pregnancy Study_F01 Screening log
    - Lassa virus in pregnancy Study_F02 Identification log
    - Lassa virus in pregnancy Study_F06 Enrolment log
    - Lassa Virus in pregnancy Study_F03 Informed Consent log
    - PIS_Lassa virus in pregnancy study_ v.1.1
    - Consent_Lassa virus in pregnancy study_ v.1.1
    - Assent_Lassa virus in pregnancy study_ v.1.1
