# Supplementary material for: Seroepidemiology of Lassa virus in pregnant women in Southern Nigeria: A prospective hospital-based cohort study
Source: PLoS Negl Trop Dis. 2023 May 22;17(5):e0011354. doi: 10.1371/journal.pntd.0011354 (PMC10237645; doi:10.1371/journal.pntd.0011354)
Supplement: S4 File — (PDF) [file pntd.0011354.s004.pdf]

CASE RECORD FORM - SEROEPIDEMIOLOGY OF LASSA VIRUS IN PREGNANCY

ENROLMENT QUESTIONNAIRE

Study Code	Participant code
LVP-OX	

INCLUSION CRITERIA	Yes	No
Pregnant women attending antenatal clinic (ANC)		
Pregnant women who are willing and able to give informed consent for participation in the study		
Aged ≥15years		
**EXCLUSION CRITERIA**	**Yes**	**No**
Legally authorised representative (LAR) is absent or unavailable		
Patients for end of life care		
Participant withdraws consent		

If the eligibility criteria have been confirmed, the individual can be enrolled in the study

ENROLMENT QUESTIONNAIRE

ENROLMENT VISIT DATES

Date of inclusion or enrolment	____/____/_______
Date of interview	____/____/_______
Name of site/clinic hospital	☐ Irrua Specialist Teaching Hospital (ISTH)<br>☐ Esan West<br>☐ Esan Central
Full Name of staff completing the questionnaire	
Signature of staff completing the questionnaire	

ENROLMENT MEDICAL DATA

Demographic Data

**1.** Date of birth (DD/MM/YYYY)<br>***If the patient doesn't know her date of birth then record this as 15/06/YYYY. The year of birth can be extrapolated from the age.***	___/____/_______	
**2.** Age	[__ | __] years	
**3.** Date of last normal menstrual period	____/____/_______	☐Certain<br>☐Uncertain ☐Unknown
**4.** Current gestational age based on LNMP	[__ |__] Weeks	
**5.** Gestational age from fundal height	[__ |__] Weeks	
**6.** Estimated date of delivery	____/____/_______	
**7.** Residence	☐ Urban<br>☐ Rural	
**8.** Area of residence in last 6 months (district name)		
**9.** Religion	☐ None<br>☐ Christian<br>☐ Muslim<br>☐ Traditional religions<br>☐ if Other religion specify:	
**10.** Educational level	☐ No formal education<br>☐ Formal education- primary<br>☐ Formal education – secondary	

ENROLMENT QUESTIONNAIRE

	☐ Formal education – tertiary
**11.** Occupation	☐ None (no profession)<br>☐ Housewife<br>☐ Healthcare Occupations (*nurse, ward assistants, doctors etc*)<br>☐ Farming and Forestry Occupations<br>☐ Education (*student/teacher at any level*)<br>☐ Office and Administrative Occupations *(banking, legal, management, public office etc*)<br>☐ Other specify
**12.** Knowledge of Lassa Fever risk factors	☐ No knowledge<br>☐ 1 or 2 risk factors<br>☐ 3 or more risk factors (good)
**13.** Knowledge of Lassa Fever transmission	☐ No knowledge<br>☐ 1 or 2 routes<br>☐ 3 or more routes (good)
**In the last 6 months has she**	
**14.** Been exposed to rodents<br>*(e,g. eating, or rats seen at home, rat burrows in house or killing rats at home)*	☐ Yes<br>☐ No
**15.** Cared for someone who was bleeding due to unknown causes	☐ Yes<br>☐ No
**16.** Participated in funeral rites (washing of the body/clothes of deceased)	☐ Yes<br>☐ No
**17.** Cared for someone who was diagnosed with Lassa fever	☐ Yes<br>☐ No
**18.** Cared for someone who died of unknown causes	☐ Yes<br>☐ No

Medical and Obstetric History *(verify with ANC and/or hospital notes)*

**19.** Number of previous pregnancies (excluding present pregnancy):	[__ |__]
**20.** Number of previous births after 22 weeks' gestation:	[__ |__]
**21.** Number of livebirths	[__ |__]

ENROLMENT QUESTIONNAIRE

**22.** Have any previous babies been premature (<37weeks of gestation)?	☐Yes<br>☐No<br>☐Unknown
**23.** Have any previous babies weighed less than 2.5kg?	☐Yes<br>☐No<br>☐Unknown
**24.** Has she been treated for Lassa fever in the past?	☐Yes<br>☐No<br>☐Unknown
**25.** Has she been diagnosed with Lassa fever during this pregnancy?	☐Yes<br>☐No<br>☐Unknown
**26.** Has she had any fevers in the last 2 weeks?	☐Yes<br>☐No<br>☐Unknown
**27.** Has she been diagnosed with any of the following chronic medical conditions **before** pregnancy? (tick all that apply)	☐ Hypertension<br>☐ Diabetes<br>☐ Other<br>If other medical complications specify?
**28.** Has she been diagnosed with gestational diabetes in this pregnancy?	☐Yes<br>☐No<br>☐Unknown
**29.** Has she been diagnosed with pregnancy –induced hypertension (PIH) in this pregnancy?	☐Yes<br>☐No<br>☐Unknown
**30.** Has she been diagnosed with pre-eclampsia in this pregnancy?	☐Yes<br>☐No<br>☐Unknown
**31.** Has she been diagnosed with eclampsia in this pregnancy?	☐Yes<br>☐No<br>☐Unknown
**32.** Are there any other pregnancy-related complications	☐Yes<br>☐No<br>☐Unknown<br>If yes specify other pregnancy related complications:
**33.** Has she been diagnosed with HIV?	☐Yes

	☐No<br>☐Unknown<br>☐ Not answered
**34.** Has she had Malaria during the current pregnancy?	☐Yes<br>☐No<br>☐Unknown
**35.** Has she been vaccinated for tetanus?	☐Yes<br>☐No<br>☐Unknown
**36.** If yes to question 26 above, number of tetanus (TT) vaccines received?	☐ 1<br>☐ 2<br>☐ 3<br>☐ 4<br>☐ 5
**37.** Please list all medication she is currently using?<br><br>__<br>*(use generic names NOT brand names)*	
**38.** On what date was the 1st (enrolment) blood sample collected from mother?<br>***(DD/MM/YYYY e.g. 01/02/2019)***	___/ ___/ ________
**39.** At what time was the 1st (enrolment) blood sample was collected from mother?<br>***(time in 24hour e.g 13:05 or 08:05)***	[__ |__ **:** ___ |____]

Ultrasound Results (*do not use old ultrasound results unless they are ≤ 1 week old*)

**40.** Was ultrasound performed?	☐Yes<br>☐No
**41.** If no why was it not performed	☐Patient refused<br>☐Patient not cooperative<br>☐Ultrasound machine not functional<br>☐Other<br>If other reason specify:
**42.** Ultrasound date	

ENROLMENT QUESTIONNAIRE

	____/____/_______
**43.** Gestational age by ultrasound	[__ |__]<br>Weeks
**44.** Fetal growth restriction	☐Yes<br>☐No<br>☐Unknown
**45.** Any other significant findings on Ultrasound	☐Yes<br>☐No<br>☐Unknown<br>If yes specify:

Has a BLOOD SAMPLE been collected for this patient?
Cross-check that all questions have been answered.
Give the patient a yellow card, explain she should call the number on the card when in labour.
