# Supplementary material for: Seroepidemiology of Lassa virus in pregnant women in Southern Nigeria: A prospective hospital-based cohort study
Source: PLoS Negl Trop Dis. 2023 May 22;17(5):e0011354. doi: 10.1371/journal.pntd.0011354 (PMC10237645; doi:10.1371/journal.pntd.0011354)
Supplement: S1 Table — (DOCX) [file pntd.0011354.s005.docx]

**S1 Table:** Factors associated with Lassa maternal IgG seropositivity at baseline.

| Factor | N | n | OR crude  [95% CI] | P crude | OR adjusted ^a^  [95% CI] | P adjusted^a^ |
| --- | --- | --- | --- | --- | --- | --- |
| **Age (years)** | 240 | 97 | 1·01[0·96 – 1·06] | 0·645 | — | — |
| **GA at enrolment^c^ (weeks)** | 234 | 92 | 1·01 [0·98 – 1·04] | 0·509 | — | — |
| **Parity** | 240 | 97 | 1·15 [0·81 – 1·62] | 0·434 | — | — |
| **Lives in a rural area** |  |  |  |  |  |  |
| No | 110 | 50 | 1·47 [0·88 – 2·48] | 0·141 | 1·40 [0·82 – 2·40] | 0·210 |
| Yes | 130 | 47 | Reference |  | Reference |  |
| **Educational level** |  |  |  |  |  |  |
| Post-secondary | 73 | 63 | Reference |  | Reference |  |
| No Post-secondary | 167 | 34 | 1·41 [0·81 – 2·45] | 0·225 | 1·21 [0·67 – 2·17] | 0·522 |
| **Occupation** |  |  |  |  |  |  |
| Student | 15 | 5 | Reference |  | — | — |
| Housewife | 39 | 18 | 1·72 [0·52 – 6·11] | 0·829 ^b^ | — | — |
| Health professional | 25 | 4 | 0·39 [0·09 – 1·69] |  | — | — |
| Informal Sector | 100 | 48 | 1·76 [0·60 – 5·70] |  | — | — |
| Formal Sector | 61 | 22 | 1·12 [0·36 – 3·77] |  | — | — |
| **Knowledge of LF** |  |  |  |  |  |  |
| Good | 30 | 9 | Reference |  | Reference |  |
| Poor | 210 | 88 | 1·66 [0·76 – 3·90] | 0·209 | 1·44 [0·58 – 3·81] | 0·431 |
| **Exposure to rodents** |  |  |  |  |  |  |
| No | 49 | 17 | Reference |  | — | — |
| Yes | 191 | 80 | 1·36 [0·72 – 2·65] | 0·342 | — | — |
| **Possible exposure to LF patients** |  |  |  |  |  |  |
| Unlikely | 226 | 94 | Reference |  | Reference |  |
| Likely | 14 | 3 | 0·42 [0·10 – 1·31] | 0·141 | 0·64 [0·15 – 2·20] | 0·486 |
| **Fever during pregnancy** |  |  |  |  |  |  |
| No | 226 | 90 | Reference |  | — | — |
| Yes | 14 | 7 | 1·49 [0·51 – 4·32] | 0·459 | — | — |
| **Positive history of Lassa fever** |  |  |  |  |  |  |
| No | 235 | 95 | Reference |  | — | — |
| Yes | 5 | 2 | 1·05 [0·17 – 5·48] | 0·956 | — | — |
| **Malaria during pregnancy^d^** |  |  |  |  |  |  |
| No | 175 | 66 | Reference |  | Reference |  |
| Yes | 64 | 30 | 1·49 [0·83 – 2·65] | 0·180 | 1·32 [0·71 – 2·41] | 0·377 |
| **Hypertension** |  |  |  |  |  |  |
| No | 235 | 95 | Reference |  | — | — |
| Yes | 5 | 2 | 1·04 [0·17 – 5·48] | 0·956 | — | — |
| **Pregnancy-induced hypertension** |  |  |  |  |  |  |
| No | 235 | 94 | Reference |  | — | — |
| Yes | 5 | 3 | 0.86 [0·17 – 3·62] | 0·849 | — | — |
| **Diabetes Mellitus**^e^ |  |  |  |  |  |  |
| No | 231 | 95 | Reference |  | — | — |
| Yes | 9 | 2 | 0·40 [0·06 – 1·71] | 0·264 | — | — |
| **HIV – infected** |  |  |  |  |  |  |
| No | 226 | 86 | Reference |  | Reference |  |
| Yes | 14 | 11 | 4·28 [1·31 – 17·5] | 0·015 | 4·24 [1·32 – 17·3] | 0·014 |

Note: For statistical significance, Bonferroni p <0.008

CI- confidence interval; GA- gestational age; LF- Lassa fever; n- number of seropositive women; N- total number of women who were enrolled into the study (240); OR- odds ratio.

^a^ Adjusted for factors which were marginally significant in the univariate regression (crude p<0·25).

^b^ Overall P value.

^c^ Data missing for 6 women.

^d^ Data missing for 1 woman.

^e^ None of the women with gestational diabetes were seropositive thus diabetes is evaluated as one group to prevent separation in the model.
