# Supplementary material for: Seroepidemiology of Lassa virus in pregnant women in Southern Nigeria: A prospective hospital-based cohort study
Source: PLoS Negl Trop Dis. 2023 May 22;17(5):e0011354. doi: 10.1371/journal.pntd.0011354 (PMC10237645; doi:10.1371/journal.pntd.0011354)
Supplement: S2 Table — (DOCX) [file pntd.0011354.s006.docx]

**S2 Table:** Characteristics of participants lost-to-follow-up (LTFU) & an estimation of the effects of LTFU

| Characteristics | N | Retained | Lost to follow up | P value^a^ |
| --- | --- | --- | --- | --- |
| **Total** | 240 | 173 (72·08) | 67 (27·91) |  |
| **Age (years) - Mean age ± sd (range)** |  | 31·56 ± 4·87 [20 - 46] | 31·58 ± 5·40 [22 - 45] | 0·982^b^ |
| **Gestational age (weeks) - Median [IQR]** |  | 24 [18 - 31] | 27 [19 - 32] | 0·239^c^ |
| **Lives in a rural area** |  |  |  |  |
| No | 110 | 77 (44·51) | 33 (49·25) |  |
| Yes | 130 | 96 (55·49) | 34 (50·75) | 0·605 |
| **Educational level** |  |  |  |  |
| Primary | 11 | 8 (4·62) | 3 (4·48) |  |
| Secondary | 62 | 35 (20·23) | 27 (40·29) | 0·007 |
| Post-secondary | 167 | 130 (75·14) | 37 (55·22) |  |
| **Occupation** |  |  |  |  |
| Student | 15 | 11 (6·36) | 4 (5·97) |  |
| Housewife | 39 | 29 (16·76) | 10 (14·93) |  |
| Health Professional | 25 | 20 (11·56) | 5 (7·46) | 0·431 |
| Informal Sector | 100 | 66 (38·15) | 34 (50·74) |  |
| Formal Sector | 61 | 47 (27·17) | 14 (20·89) |  |
| **Parity** |  |  |  |  |
| 0 | 50 | 36 (20·81) | 14 (20·89) |  |
| 1 – 2 | 98 | 73 (42·19) | 25 (37·31) | 0·749 |
| ≥ 3 | 92 | 64 (36·99) | 28 (41·79) |  |
| **Knowledge of LF risk factors & transmission** |  |  |  |  |
| Good | 30 | 22 (12·72) | 8 (11·94) |  |
| Poor | 210 | 151 (87·28) | 59 (88·06) | 1 |
| **Exposure to rodents** |  |  |  |  |
| No | 49 | 34 (19·65) | 15 (22·39) |  |
| Yes | 191 | 139 (80·35) | 52 (77·61) | 0·721 |
| **Possible exposure to LF patients** |  |  |  |  |
| Unlikely | 226 | 161 (93·06) | 65 (97·01) |  |
| Likely | 14 | 12 (6·93) | 2 (2·99) | 0·361 |
| **Fever during pregnancy** |  |  |  |  |
| No | 198 | 139 (80·35) | 59 (88·06) |  |
| Yes | 42 | 34 (19·65) | 8 (11·94) | 0·222 |
| **History of Lassa fever** |  |  |  |  |
| No | 235 | 169 (97·69) | 66 (98·51) |  |
| Yes | 5 | 4 (2·31) | 1(1·49) | 1 |
| **Malaria during pregnancy** |  |  |  |  |
| No | 143 | 104 (60·12) | 39 (58·21) |  |
| Yes | 97 | 69 (39·88) | 28 (41·79) | 0·884 |
| **Hypertension** |  |  |  |  |
| No | 227 | 163 (94·21) | 64 (95·52) |  |
| Yes | 13 | 10 (5·78) | 3 (4·48) | 1 |
| **Diabetes Mellitus** |  |  |  |  |
| No | 231 | 167 (96·53) | 64 (95·52) |  |
| Yes | 9 | 6 (3·47) | 3 (4·48) | 0·712 |
| **HIV - infected** |  |  |  |  |
| No | 226 | 165 (95·38) | 61 (91·04) |  |
| Yes | 14 | 8 (4·62) | 6 (8·96) | 0·224 |
| **Lassa serostatus at baseline** |  |  |  |  |
| Equivocal | 2 | 2 (1·2) | 0 |  |
| Negative | 141 | 105 (60·6) | 36 (53·7) | 0·423 |
| Positive | 97 | 66 (38·2) | 31 (46·3) |  |

Note: Data are presented as n (%) as applicable, where n is the number of pregnant women; N- is total number of pregnant women; LF- Lassa fever.

^a^ χ² ^or^ Fischer’s exact p value.

^b^ Student t-test p value.

^c^ Mann-Whitney U p value and data missing for 6 women.
